# Dr. Hiram Gettman Du Bois

**Published:** 1911-11

**Authors:** 


Dr. Hiram Gettman Du Bois, University of Buffalo, 1866, once president and twice vice-president of the Medical Society of the County of Oneida, died at Camden, September 19, 1911, of cerebral hemorrhage.

